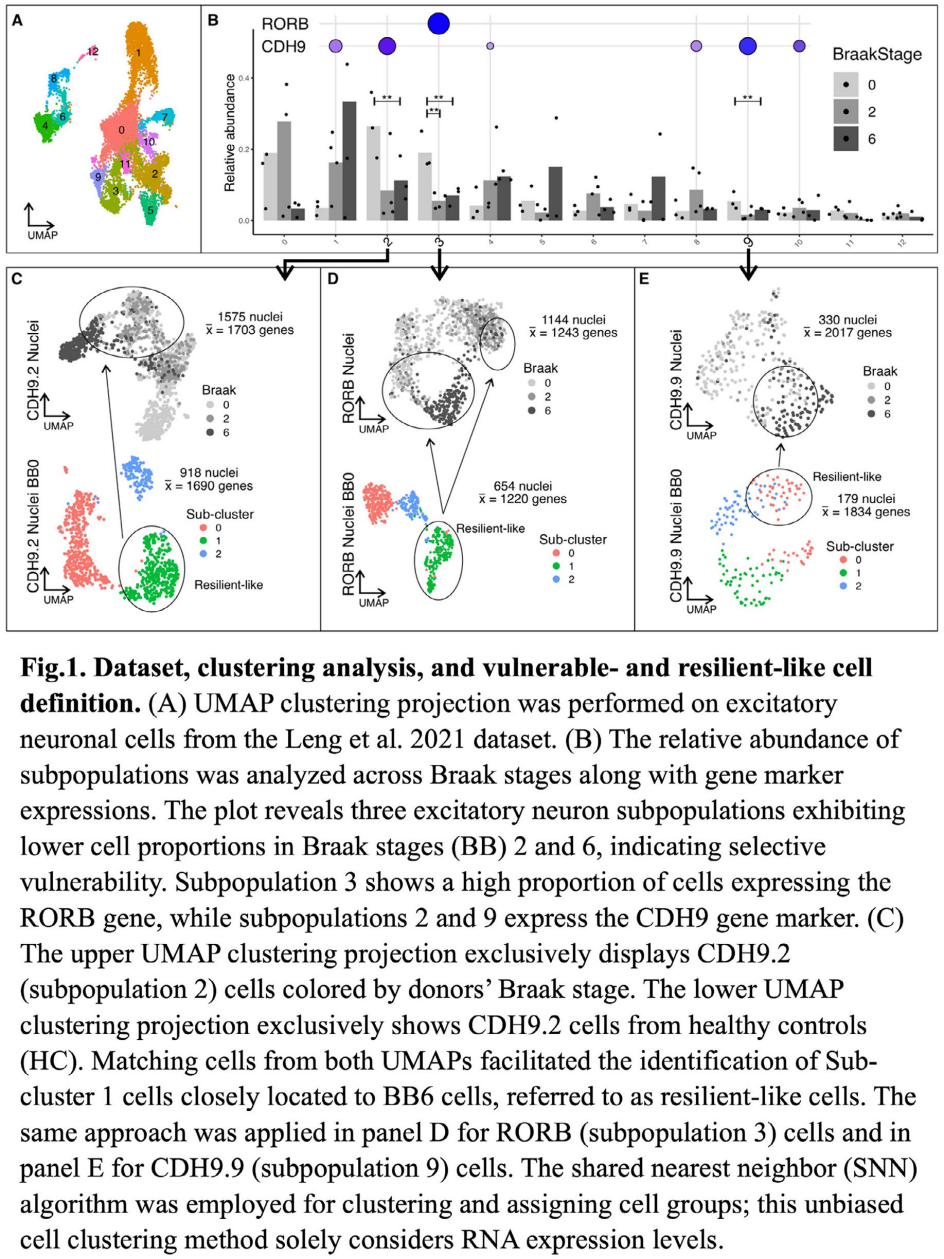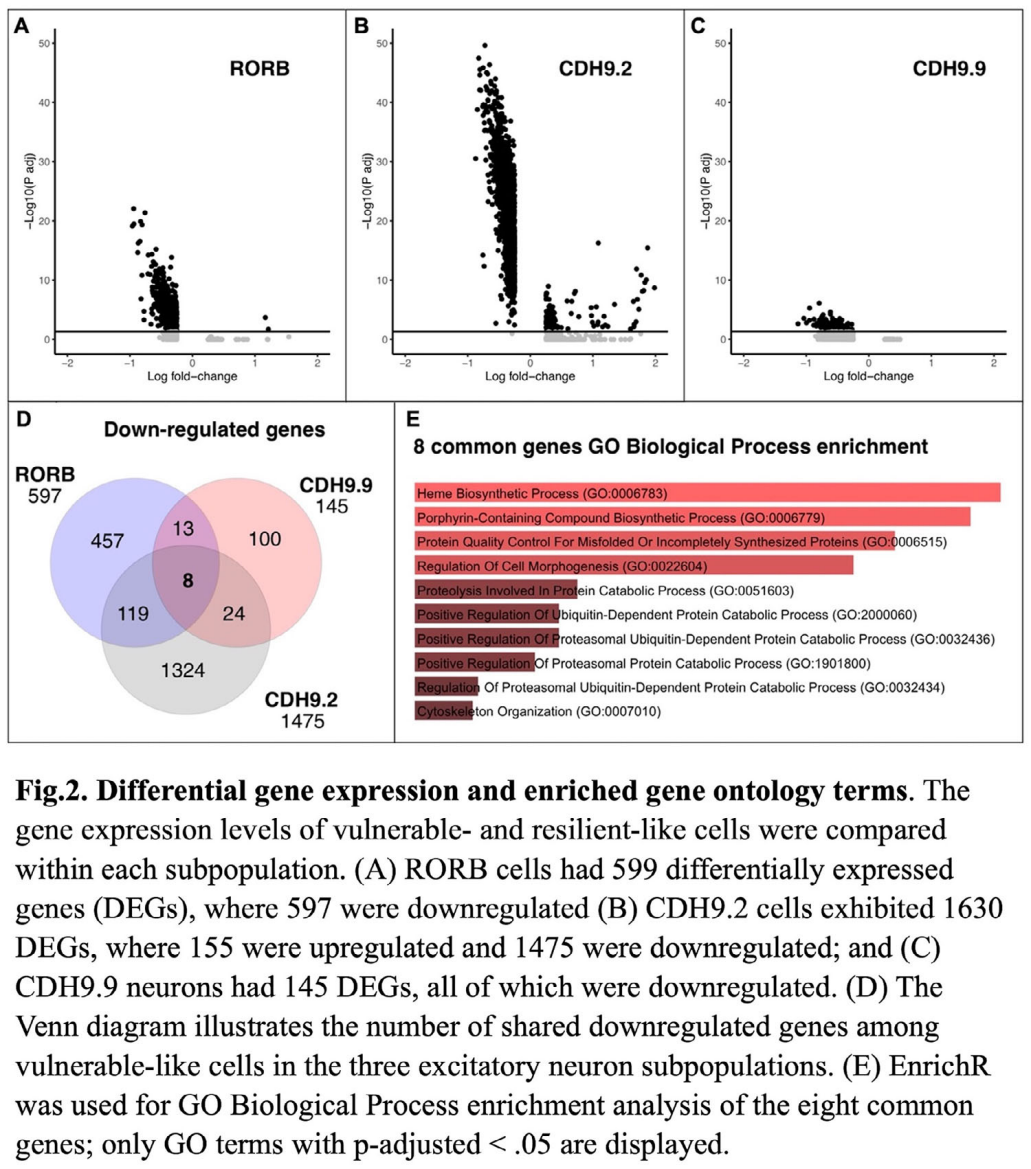# Exploring selective neuronal vulnerability in Alzheimer’s disease – key pathways are downregulated in vulnerable subpopulations of RORB and CDH9 excitatory neurons of the entorhinal cortex at Braak 0

**DOI:** 10.1002/alz.091740

**Published:** 2025-01-03

**Authors:** Alexander V. Soloviev, Felipe Luiz Pereira, Renata Elaine Paraizo Leite, Claudia Kimie Suemoto, Kun Leng, Martin Kampmann, Lea T. Grinberg

**Affiliations:** ^1^ Memory and Aging Center, UCSF Weill Institute for Neurosciences, University of California San Francisco, San Francisco, CA USA; ^2^ Physiopathology in Aging Laboratory (LIM‐22), University of São Paulo Medical School, São Paulo, São Paulo Brazil; ^3^ Division of Geriatrics, University of São Paulo Medical School, São Paulo, São Paulo Brazil; ^4^ UCSF School of Medicine, University of California San Francisco, San Francisco, CA USA; ^5^ Department of Biochemistry and Biophysics, Weill Institute for Neurosciences, University of California San Francisco, San Francisco, CA USA; ^6^ Memory & Aging Center, Department of Neurology, University of California in San Francisco, San Francisco, CA USA

## Abstract

**Background:**

Understanding the molecular mechanisms underlying selective neuronal vulnerability is crucial for developing effective treatments for Alzheimer’s disease (AD). Our group has shown that RORB/CDH9‐positive excitatory neurons in the entorhinal cortex (EC) display selective vulnerability as early as Braak stage (BB) 2. However, not all RORB/CDH9‐positive neurons are vulnerable. By further leveraging single‐nucleus RNA sequencing (snRNA‐seq) data, we aimed to identify molecular signatures of selective neuronal vulnerability. This involved comparing resilient‐like subpopulations (BB0 cells with RNA expressions similar to BB6) and vulnerable‐like subpopulations (BB0 cells with different RNA expressions than BB6) within each RORB and CDH9 subpopulation.

**Method:**

Bioinformatic analyses were conducted using the Seurat R library on a published snRNA‐seq dataset of isolated nuclei extracted from the EC of postmortem brain tissue from healthy controls and AD patients (BB0 [n = 3], BB2 [n = 4], and BB6 [n = 3]) (Fig. 1). The Wilcox statistical test (p‐adjusted <0.05) was applied throughout the FindMarkers function to identify differentially expressed genes (DEGs) on vulnerable‐ and resilient‐like cells.

**Result:**

From the original dataset, with EC excitatory neurons (11488 nuclei), we found one RORB‐ and two CDH9‐positive (CDH9.2 and CDH9.9) vulnerable‐like neuronal subpopulations (Fig. 1). In RORB neurons, downregulated genes are involved in insulin signaling, autophagy, and ErbB signaling pathways (Fig. 2A). In CDH9.2 neurons downregulated genes are involved in membrane trafficking, axon guidance, proteasome‐mediated ubiquitin‐dependent protein catabolic process, and protein polyubiquitination (Fig. 2B). Additionally, in CDH9.9 neurons downregulated genes are involved in membrane trafficking, Ras protein signal transduction, GTPase activator activity, and ubiquitin‐like protein ligase binding (Fig. 2C). These three susceptible subpopulations share eight downregulated genes, some of which are highly involved in the Heme Biosynthetic Process. Heme is a common element linking various metabolic disruptions in AD, including oxygenase, mitochondrial dysfunction, and inhibition of muscarinic acetylcholine receptor binding.

**Conclusion:**

Our analyses suggest that changes in metabolic, ubiquitin‐proteasome, and cell growth/survival pathways occur in vulnerable‐like cells of the EC in BB0. Therefore, these pathways may be potential therapeutic targets in the earliest stages of AD. By looking into younger cohorts, we will further investigate if the molecular differences between these neuronal subpopulations are canonical (indicating a pathological predisposition) or an early pathological response.